# Study on Hot Deformation Behavior of an Antibacterial 50Cr15MoVCu Tool Steel

**DOI:** 10.3390/ma15103460

**Published:** 2022-05-11

**Authors:** Ziyuan Liu, Zhao Yang

**Affiliations:** School of Materials Science and Engineering, Central South University, Changsha 410083, China; 8204190418@csu.edu.cn

**Keywords:** 50Cr15MoVCu steel, hot deformation behavior, flow curve, constitutive equation, processing map

## Abstract

Hot deformation behaviors of an antibacterial 50Cr15MoVCu tool steel were studied. The flow stress curves presented three typical characteristics: (i) a single peak dynamic recrystallization curve, (ii) a monotone incremental work-hardening curve, and (iii) the equilibrium dynamic recovery curve. The flow stress increased with the increase of the deformation rate at each deformation temperature and decreased with the increase of the deformation temperature at the same deformation rate. The thermal activation energy and material constants were Q of 461.6574 kJ/mol, A of 3.42 × 10^17^, and α of 0.00681 MPa^−1^, respectively. The high temperature constitutive equation was: $Z=\dot{\varepsilon }\exp\left(Q/\mathrm{RT}\right)=3{.42\;\times \;10}^{17}{\left[\sinh\left(0.0068\;\times \;\sigma \right)\right]}^{5.6807}$. Based on the processing maps and microstructure evolution, the best hot working process was a deformation temperature of 1050 °C and deformation rate of 0.001 s^−1^.

## 1. Introduction

50Cr15MoV martensitic stainless steel has been widely used as kitchen knives due to its high strength, hardness, and good corrosion resistance [1]. With the improvement of living standards, high-grade kitchen knives are great attractive and not only beautiful but also antibacterial. The antibacterial ability of metals can be increased by alloying with an antimicrobial element such as copper (Cu) [2,3,4]. Cu has good antibacterial and antifungal effects [5]. Sun et al. [2] demonstrated that, after 4.5 wt% copper was alloyed into 317L stainless steel (317L SS), the obtained 317L-Cu SS showed strong antibacterial efficacy, having a 99% inhibition rate of sessile Staphylococcus aureus cells after 5 days. Guo et al. [3] found that Fe-xCu alloys produced by selective laser melting presented a strong antibacterial performance with antibacterial rates against *E. coli* up to 99.9%. Ji et al. [4] documented that Ti-xCu alloys prepared by selective laser melting had satisfactory antibacterial properties with antibacterial rates against *E. coli* up to 99.9%. The antibacterial mechanism of the above various Cu-containing alloys was attributed to the release of Cu ions from the corresponding alloys [5,6]. Based on the antibacterial functions of Cu, an idea is to prepare Cu-containing 50Cr15MoV steel, in which Cu is functionalized as an antibacterial activity. In this way, a desired antibacterial effect should be obtained for 50Cr15MoVCu steel. Therefore, 50Cr15MoVCu steel is a promising type of antibacterial steel for kitchen knives application.

However, Cu needs to be controlled in the traditional alloy design and manufacturing process, because it can cause the thermal embrittlement of the alloy [7]. Under the oxidizing atmosphere of more than 1083 °C that is the Cu melting point, the solubility of oxygen in liquid copper is very high, and oxygen diffuses and reacts with Cu to form cuprous oxide. Iron reacts with the oxygen of cuprous oxide, and Cu is reduced to liquid elemental Cu, because the affinity of iron to oxygen is much higher than that of Cu to oxygen. The elemental copper tends to segregate at the grain boundaries, and accordingly, grain boundary embrittlement occurs. Hot working processing plays an important role in steel production, which requires not only dimensional accuracy but also specified microstructures and mechanical properties [8]. It is necessary to control the heating temperature, rolling time, and pass reduction in the hot working processing to avoid the occurrence of hot brittleness for 50Cr15MoVCu steel, which needs to study the deformation mechanism and microstructure evolution mechanism. The processing map developed from the flow data of the thermal compression test is very important to the optimization of the processing conditions, as well as the revelation of the influence of the hot processing parameters on microstructure evolution [9]. However, there is no published literature to study the hot deformation behavior of 50Cr15MoVCu steel with a high copper content or to propose a solution to the hot embrittlement at present, which is also the innovation of this study.

A previous study showed that the cracking failure phenomenon of a kitchen knife made by 5Cr15MoV steel was mainly due to the organizational stress caused by carbide segregation in the steel. The overhigh hardness and substandard chemical composition were the secondary causes of cracking [10]. Another analysis showed that the reason for the edge crack of a 304Cu hot rolled strip was the unstable deformation of a high-temperature structure between the Cr-enriched high-temperature ferrite and austenite matrix, and the uncoordinated deformation of the high-temperature structure leads to edge cracks. In addition, the enrichment of Cu reduces the intergranular adhesion, promotes the generation of internal holes, and then increases the probability of edge crack defects [11]. In this work, the thermal deformation behavior of 50Cr15MoVCu steel and the influence of the thermal deformation parameters on the flow stress and microstructure were studied through a thermal compression simulation experiment, and the optimum processing conditions for 50Cr15MoVCu steel were confirmed via the flow stress curve, constitutive equation, hot working energy dissipation diagram, hot working diagram, and microstructure analysis. The results will be beneficial to the practical application of the manufacturing processing of the production of high-grade kitchen knives with an antibacterial function.

## 2. Experimental Procedure

The chemical composition of the 50Cr15MoVCu steel in this work is shown in Table 1. The general idea of designing 50Cr15MoVCu was according to GB/T 4237-2015 (hot rolled stainless steel plate, sheet, and strip). The content of Cr was increased to improve the corrosion resistance to meet the kitchen circumstances, and the content of the antimicrobial element Cu was around 3.5% to obtain good antibacterial properties, while the content of C was controlled offline at 0.46% due to the austenitic phase form element Cu addition and the contents of the other elements, such as Si, Mn, Mo, and V were kept at the midline. In order to improve the mechanical behavior, the Cr and Ni equivalents were controlled to obtain a single martensitic microstructure.

**Figure 1:**
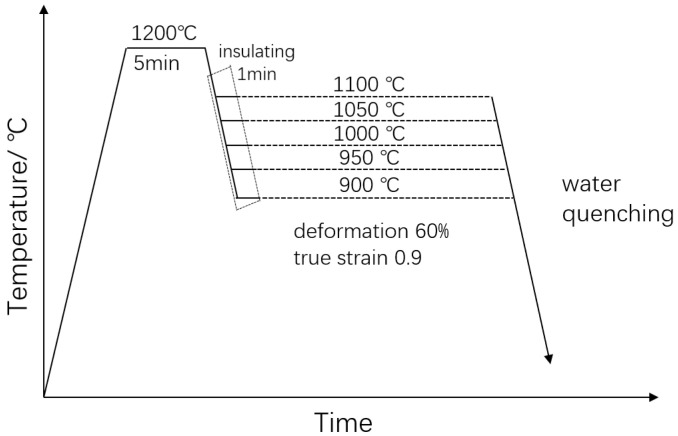
Schematic experimental procedures of hot deformation.

The steel was melted in a vacuum induction-melting furnace and then forged into a bar with diameter of 22 mm at 1200 °C. The specimens for the compression test were cut from the center of the bar and machined into dimensions of 8 mm in diameter and 12 mm in gauge length. The compression tests were conducted on a Gleeble-3800 thermal-mechanical simulator to study the deformation characteristics of the experimental steel, in which temperatures at 900, 950, 1000, 1050, and 1100 °C we chosen, and the strain rates were chosen as 0.001, 0.01, 0.1, 1, and 10 s^−1^, respectively. In order to ensure a uniform deformation and minimize the frictional effect, a tantalum foil with a thickness of 0.05 mm was introduced between the indenter and transverse surface of the specimen. Pure nickel and graphite were applied between the tantalum sheet and the specimen to prevent melting and improve the conductivity of the specimen during deformation. All the specimens were homogenized in Ar gas at 1200 °C for 5 min to eliminate the effect of the precipitations formed during solidification and forging on the deformation and to ensure the microstructure consistency of each group of specimens. The homogenized specimens were cooled at 10 °C/s to the designed deformation temperatures for 1 min and were compressed using the designed strain rate at a true strain of 0.9 (~60% height reduction), followed by water quenching to room temperature immediately to preserve the as-deformed microstructure. The procedures are schematically illustrated in Figure 1.

**Figure 2:**
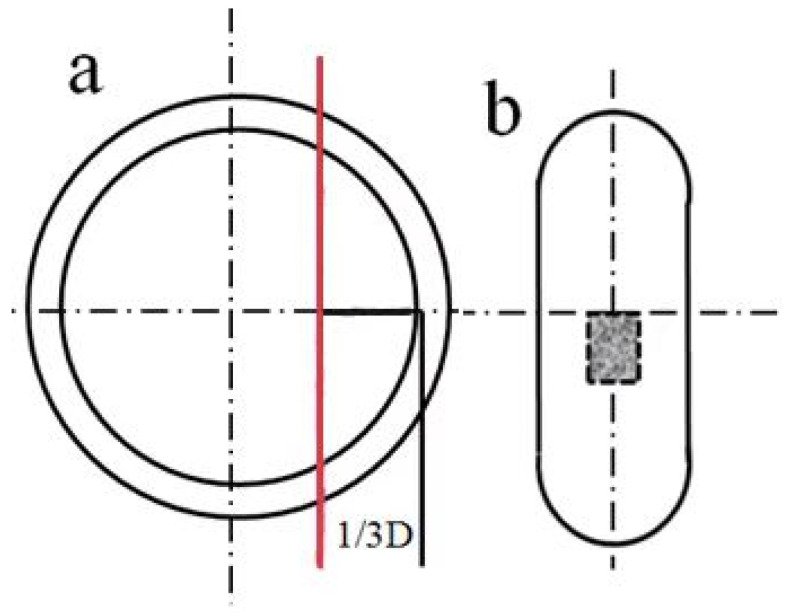
Microstructure sampling and observation location. (**a**) Vertical view of the sample after compression; the sample was cut along the red line. (**b**) Central position of the section.

The high-temperature constitutive equation was constructed based on the relationship between the stress, strain, strain rate, and temperature that was fitted by the data of the critical value at which dynamic recrystallization occurred. The critical value was deduced from the thermal deformation results using a differential function. Compressed specimens were cut at ~1/3 of the diameter from the edge along the direction of the hot compression to ensure the accuracy and stability of the thermal deformation, as schematically illustrated in Figure 2. The section was ground on waterproof SiC papers from 150 to 2000 grit in sequence and polished with a diamond paste (2.5 um) to a mirror finish, followed by etching with a reagent of H_2_O (10 mL) + HCl (4 mL) + HNO_3_ (4 mL). Then, the microstructures of the middle shadow area were observed by an optical microscope (Axiovert 200 MAT; Zeiss, Oberkochen, Germany).

## 3. Results and Discussion

### 3.1. Flow Stress Line

**Figure 3:**
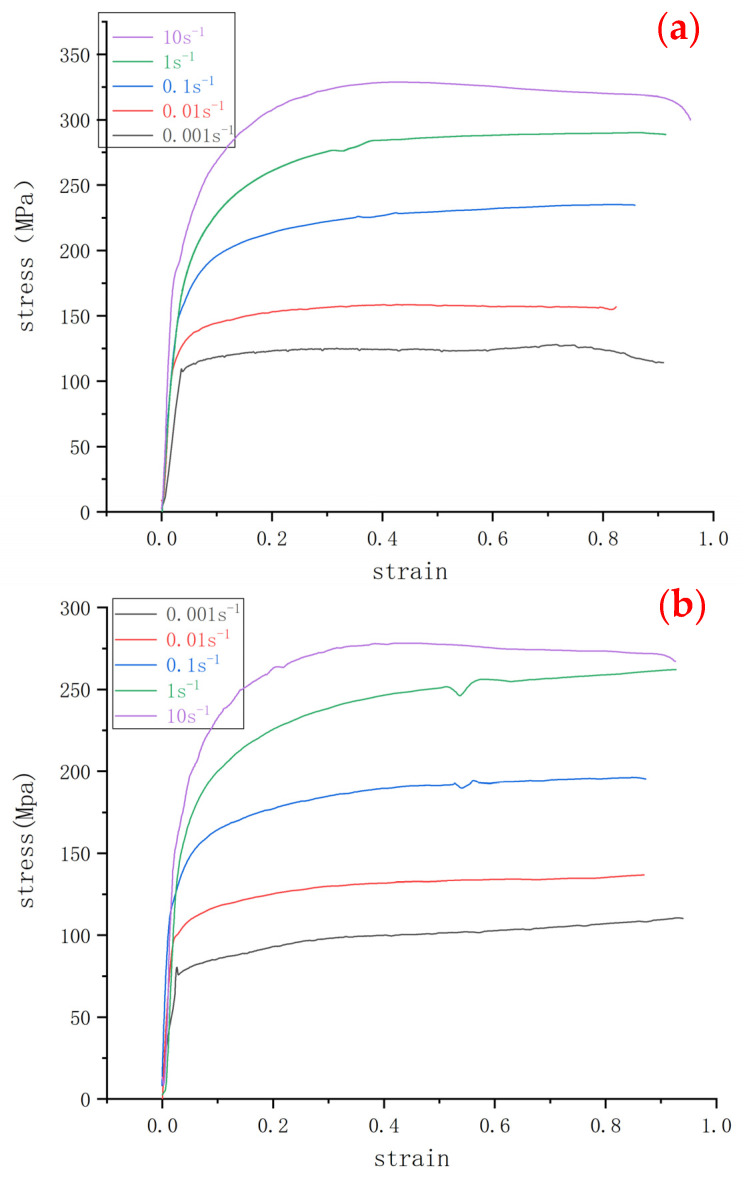

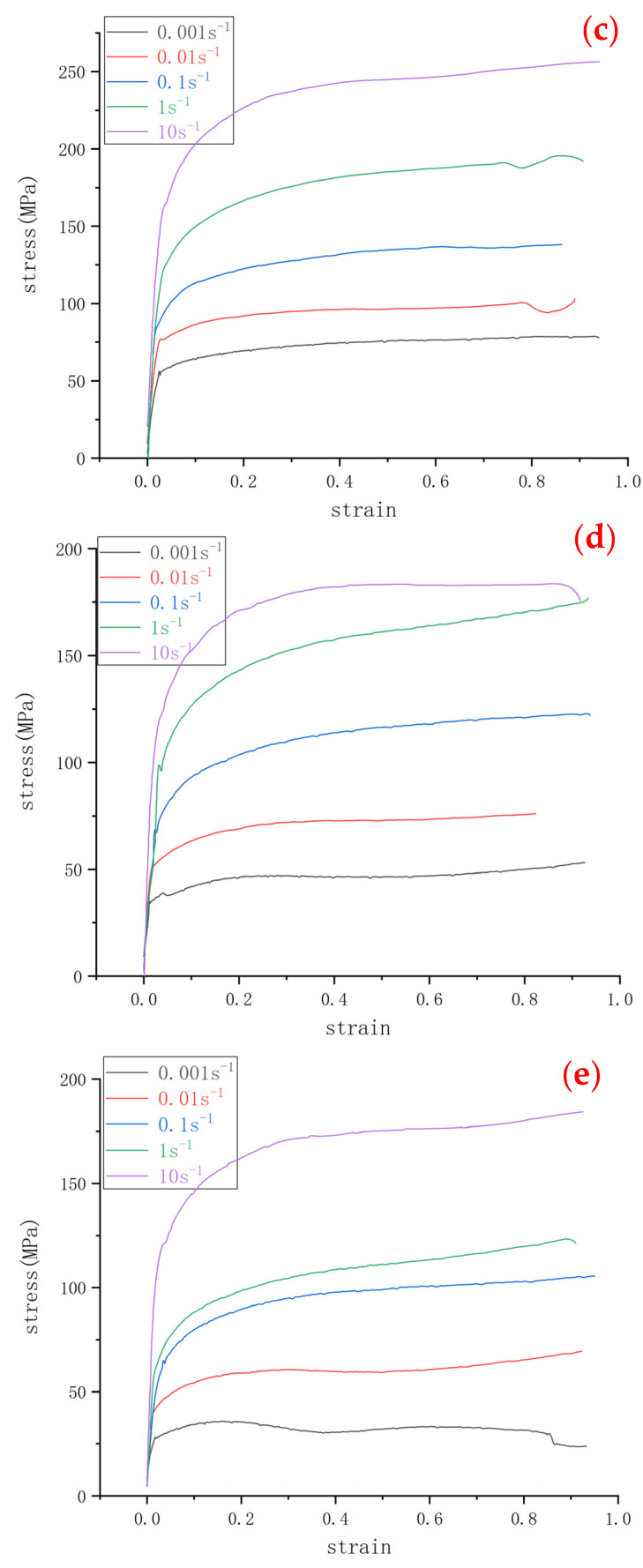
True stress-strain curves under different strain rates at each deformed temperature: (**a**) 900 °C, (**b**) 950 °C, (**c**) 1000 °C, (**d**) 1050 °C, and (**e**) 1100 °C.

Figure 3 depicts the true stress–strain curves under different strain rates and different temperatures. All curves presented a work-hardening phenomenon. The flow stress increased with the increase of the deformation rate at each deformation temperature, which might be attributed to the following mechanism, i.e., the higher the deformation rate, the shorter the deformation time, and thus, the dislocation recovery was not sufficient, and the work-hardening was predominant. Moreover, the flow stress decreased with the increase of the deformation temperature at the same deformation rate. 

All flow stress curves showed three typical characteristics, as reported [12]: (i) a single peak dynamic recrystallization curve, (ii) a monotone incremental work-hardening curve, and (iii) the equilibrium dynamic recovery curve. Flow curves at a high temperature (1100 °C) and the strain rate of 0.001 s^−1^ had a peak. The stress increased rapidly with the increase of the strain at the initial stage of deformation, reached a peak, and then decreased to a stable state. This clearly confirmed the occurrence of dynamic recrystallization (DRX). Flow curves at a high temperature (higher than 900 °C) and a strain rate of 1 s^−1^ had a monotonous increase. The stress increased rapidly with the increase of the strain at the initial stage of deformation and then increased slowly after reaching a certain critical value at which dynamic recovery (DRV) occurred. The increase in the stress could be attributed to the dominant effect of the work-hardening mechanism over the softening mechanism taking place, such as DRX and DRV [13]. Flow curves at a high temperature (1000 °C) and a strain rate of 0.1 s^−1^ firstly increased rapidly and then tended to be gentle and flat. There was no obvious peak stress on the curves, which should be attributed to the equilibrium between work hardening and DRV [13,14]. 

Moreover, the flow stress and the deformation temperature presented a negative correlation, i.e., the lower flow stress corresponded to the higher deformed temperature at the same deformation rate, as shown in Figure 3a,d as an example. As the temperature increased, the grain boundary movement accelerated, which resulted in the acceleration of the DRX. Therefore, DRX nucleation became easier at high temperatures, and the initial strain of DRX decreased. As a result, the peak strain and steady strain decreased. For example, at 1100 °C and 0.001 s^−1^, the movement capacity of the dislocation increased, the number of recrystallization nucleation increased, and accordingly, a typical DRX curve was presented.

### 3.2. High-Temperature Constitutive Equation

Reasonable hot processing technology can significantly reduce the porosity, shrinkage, segregation, and cracks, which often happen in the processing of metals. A constitutive equation that describes the relationship among the temperature, deformation rate, and flow stress can be used to predict the stress–strain behavior in hot processing. To date, the constitutive equation obtained from the hyperbolic sinusoidal formula modified by Sellar and Tegart [15] has been widely used in the field of hot processing. The expressions under different deformation conditions are as follows [15]:

(1) $${\;\dot{\varepsilon }=A}_{1}\sigma^{n_{1}}\exp(-Q/\mathrm{RT})$$

(2) $${\;\dot{\varepsilon }=A}_{2}\exp\left(\beta \sigma \right)\exp(-Q/\mathrm{RT})$$

(3) $$\;\dot{\varepsilon }=A{\left[\sinh\left.\left(\alpha \sigma \right)\right]\right.}^{n}\exp(-Q/\mathrm{RT})$$

where $\dot{\varepsilon }$ is the deformation rate; σ is the flow stress; A, $A_{1}$, $A_{2}$, $n_{1}$, n, β, and α (β/α) are the temperature-independent constants; T is the temperature (K); R is the gas constant (8.314 J·mol^−1^·K^−1^); and Q is the deformation activation energy. The above three constitutive equations can be used for thermal deformation under different conditions. Especially, Equation (3) is a hyperbolic sine function that can be used for all stress conditions. Moreover, the Z-parameter method proposed by Zener and Hollomon [16] can reflect well the relationship among the flow stress, temperature, and deformation rate, which can be expressed as follows:

(4) $$Z=\dot{\varepsilon }\exp\left(Q/\mathrm{RT}\right)$$

Equation (3) is substituted into Equation (4), and the following expression is obtained:

(5) $$Z=\dot{\varepsilon }\exp\left(Q/\mathrm{RT}\right)=A{\left[\sinh\left(\alpha \sigma \right)\right]}^{n}$$

The peak stress–strain values under different conditions are recorded, and the constitutive equation is obtained by solving each of its parameters. The specific calculation process is described as follows: (i) Equation (5) is transformed into a linear function; (ii) the peak stress, peak strain, and stress value under a specific strain are substituted into the linear function that has different parameters after deformation, and the slope and intercept of the curve after the fitting are recorded; and (iii) the obtained slope and intercept are substituted to obtain the final constitutive equation. 

Equations (1) and (2) are taken as the natural logarithms, and the following formulas are obtained:

(6) $$n_{1}\ln\;\sigma =\ln\;\dot{\varepsilon }-\ln\;A_{1}-Q/\mathrm{RT}$$

(7) $$\beta \sigma =\ln\;\dot{\varepsilon }-\ln\;A_{2}+Q/\mathrm{RT}$$

**Figure 4:**
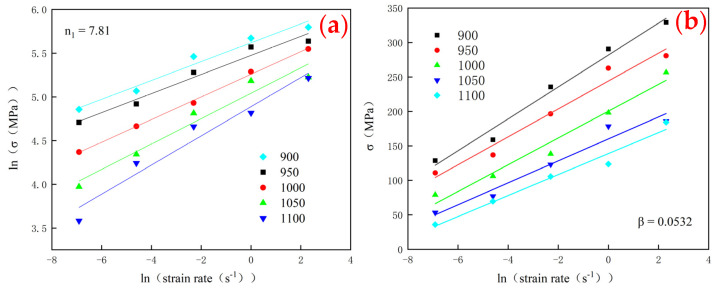

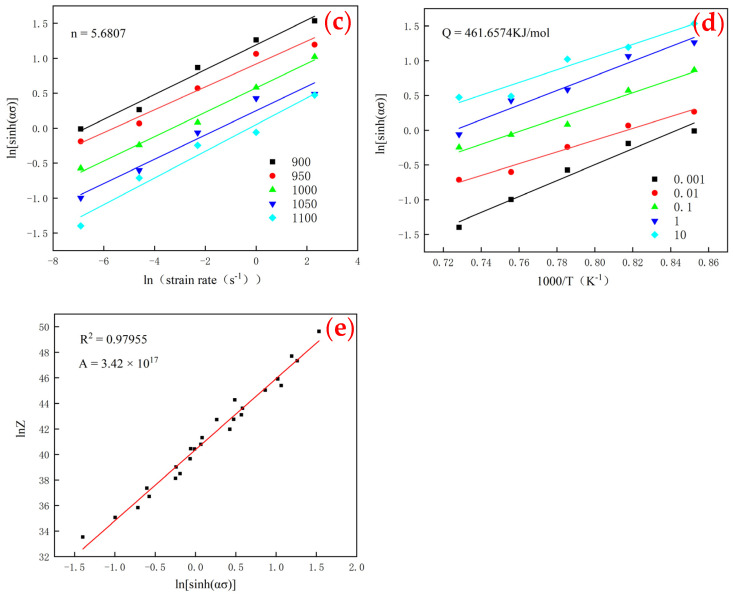
Fitting map of each parameter for the constitutive equation. (**a**) ln σ-ln $\;\dot{\varepsilon }$ and fitting of $n_{1}$, (**b**) σ-ln $\dot{\varepsilon }$ and fitting of β, (**c**) $\ln\left[\sinh\left(\alpha \sigma \right)\right]-\ln\;\dot{\varepsilon }$ and fitting of n, (**d**) $\ln\left[\sinh\left(\alpha \sigma \right)\right]-1/T$ and fitting of Q, (**e**) $\ln\left[\sinh\left(\alpha \sigma \right)\right]-\mathrm{lnZ}$, fitting of A and the correlation coefficient (R^2^) of the parameters.

As shown in Figure 4a,b, the parameters $n_{1}$ and β are obtained by drawing ln σ - ln $\dot{\varepsilon }$ and σ - ln $\dot{\varepsilon }$. The average values of $n_{1}$ and β are 7.806 and 0.0532, respectively, which are fitted by the least square method. As a consequence, the stress factor α, which is equal to β/$n_{1}$, is 0.00681 MPa^−1^. 

Equation (3) is taken as the natural logarithm, and the following formula is obtained:

(8) $$\ln\left[\sinh\left(\alpha \sigma \right)\right]=\frac{\ln\;\dot{\varepsilon }}{n}-\frac{\ln\;A}{n}+\frac{Q}{\mathrm{nRT}}$$

As shown in Figure 4c, the average value of the stress index n is obtained as 5.6807 by drawing $\ln\left[\sinh\left(\alpha \sigma \right)\right]-\ln\;\dot{\varepsilon }$. Similarly, Q is obtained as 461.6574 kJ/mol by fitting $\ln\left[\sinh\left(\alpha \sigma \right)\right]-1/T,$ as shown in Figure 4d, and A is obtained as 3.42 × 10^17^ by fitting $\ln\left[\sinh\left(\alpha \sigma \right)\right]-\ln\;Z$, as shown in Figure 4e. All the calculated parameters are substituted into Equation (5), and the constitutive equation is obtained as follows:

(9) $$Z=\;\dot{\varepsilon }\exp\left(Q/\mathrm{RT}\right)=3{.42\;\times \;10}^{17}{\left[\sinh\left(0.0068\;\times \;\sigma \right)\right]}^{5.6807}$$

Based on the Arrhenius theory, the activation energy Q of the thermal deformation represents the critical value required by atomic diffusion. In the deformation of most metals at a high temperature (>0.5 T_m_), the fluctuation of Q is strongly influenced by the solid solution strengthening of alloying elements, the occurrence of dynamic recrystallization, the induced precipitation of a precipitated phase, and phase transformation. In this work, the Q value of the 50Cr15MoVCu tool steel is 461.6574 kJ/mol, much higher than the self-diffusion activation energy of γ-Fe (280 kJ/mol [17]), and accordingly, dynamic recrystallization and dynamic recovery are the main mechanisms.

### 3.3. Thermal Processing Maps

The thermal processing maps can be applied to optimize the hot workability and control the microstructure in a wide temperature and strain rate regimes for hot working. It is of great significance to build a thermal processing map that can intuitively evaluate the thermal processing properties of metals and reflect the rationality of the thermal deformation parameters. At present, the main model of the thermal processing map is the dynamic material model (DMM), and the obtained thermal processing map includes a power dissipation diagram and rheological instability diagram [18].

According to the DMM, when the deformation temperature is constant, the relationship between the flow stress and strain rate of the experimental steel in the hot compression process is described by [19]:

(10) $${\sigma =K\;\dot{\varepsilon }}^{\;m}$$

where K is a constant, σ is the flow stress, $\dot{\varepsilon }\;$is the strain rate, and m is the strain rate sensitivity coefficient. 

Equation (10) is a basic dissipation relation. According to the dissipative structure theory, the total energy P given externally can be divided into two parts during thermal deformation: (i) the energy is lost in the form of heat during deformation, identified as G, and (ii) the energy is consumed for the internal microstructure evolution, identified as J. The relationship among P, J, and G is expressed as follows [19]:

(11) $$G=\int_{0}^{\;\dot{\varepsilon }}\sigma d\dot{\varepsilon }$$

(12) $$J=\int_{0}^{\sigma }\dot{\varepsilon }d\sigma$$

(13) $$P=\sigma \times \;\dot{\varepsilon }=G+J=\int_{0}^{\;\dot{\varepsilon }}\sigma d\dot{\varepsilon }+\int_{0}^{\sigma }\dot{\varepsilon }d\sigma$$

When the deformation temperature and strain rate are constant, J can be obtained by combining Equations (10) and (13):

(14) $$J=\frac{m}{m+1}P=\frac{m}{m+1}\sigma \;\dot{\varepsilon }$$

Based on Equations (13) and (14), the relative values of G and J can be determined by m as follows:

(15) $$m=\frac{J}{G}=\frac{\partial J}{\partial G}=\frac{\;\dot{\varepsilon }\partial \sigma }{\sigma \partial \;\dot{\varepsilon }}=\frac{\partial \left(\ln\sigma \right)}{\partial \left(\ln\;\dot{\varepsilon }\right)}$$

When m ranges from 0 to 1, the metal is in a dissipative state. When m is 1, half the total input energy is used in the microstructural change, which is an ideal state, and J reaches the peak value:

(16) $$J_{\mathrm{Max}}=\sigma \;\dot{\varepsilon }/2$$

**Figure 5:**
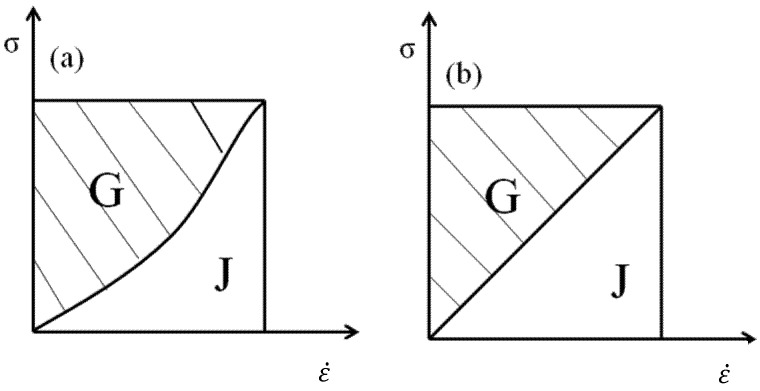
Material system energy dissipation map. (**a**) Nonideal energy dissipation map. (**b**) ideal energy dissipation map.

Figure 5 shows the energy dissipation map of the material system, in which the blank area below the curve represents J, and the shadow area above the curve represents G [20].

The energy dissipation efficiency factor, ƞ, which is the dimensionless parameter value and is derived from the ratio of Equations (14)–(16) is defined as the ratio of the energy consumed by microstructural evolution in the thermal processing process to the energy in the ideal state [21]. That is:

(17) $$\eta =\frac{J}{J_{\mathrm{Max}}}=\frac{2m}{m+1}$$

Therefore, ƞ is closely related to m, which depends on the deformation temperature, strain rate, and deformation amount. The value of ƞ under different conditions can be calculated by substituting the m value, and thus, the energy dissipation diagram can be obtained. The solution of the m value is described as follows. 

During thermal deformation, the relationship between ln σ and ln $\dot{\varepsilon }=A$ at a constant temperature is expressed as:

(18) $$\ln\sigma =a+\mathrm{bln}\;\dot{\varepsilon }+c(\ln\;\dot{\varepsilon }{)}^{2}+d(\ln\;\dot{\varepsilon }{)}^{3}$$

In which a, b, c, and d can be obtained by fitting the data at 900, 950, 1000, 1050, and 1100 °C, respectively. Therefore, the m value under different temperatures and strain rates can be obtained by combining Equations (12) and (16):

(19) $$m=b+2\mathrm{cln}\;\dot{\varepsilon }+3d(\ln\;\dot{\varepsilon }{)}^{2}$$

**Figure 6:**
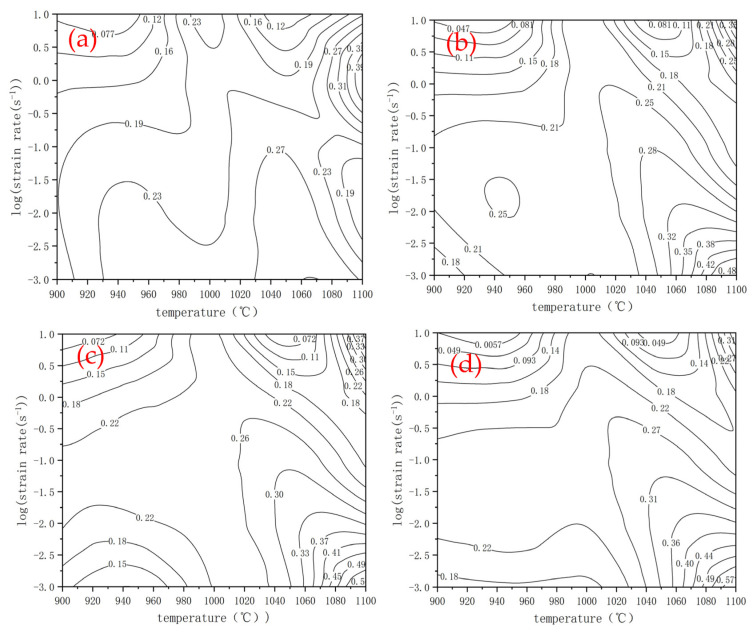
Energy dissipation map under different true strains: (**a**) 0.1, (**b**) 0.2, (**c**) 0.3, and (**d**) 0.4.

Figure 6 shows the energy dissipation diagrams. Usually, higher ƞ values represent more energy in theory applied to microstructure evolution, more microstructural stability, and a better hot working performance. The highest ƞ, however, does not necessarily reflect the best hot working range, because instability phenomenon such as cracks, adiabatic shear bands, and local deformation may occur in high dissipation areas [22]. It is necessary to determine the instability zones in hot processing so as to exclude the regions with a high ƞ but in an unstable state. 

Based on the extreme value principle of irreversible thermodynamics, Ziegler H et al. proposed the continuous instability judgment of the dynamic material model [23], which can be expressed as:

(20) $$\xi =\frac{\partial \ln\left[m/\left(m+1\right)\right]}{\partial \ln\;\dot{\varepsilon }}+m\leq 0$$

When ξ is less than 0, the material is in the instability zone of thermal processing. When ξ is more than 0, the material is in the safety zone of thermal processing, and finally, the thermal processing diagrams are obtained. 

**Figure 7:**
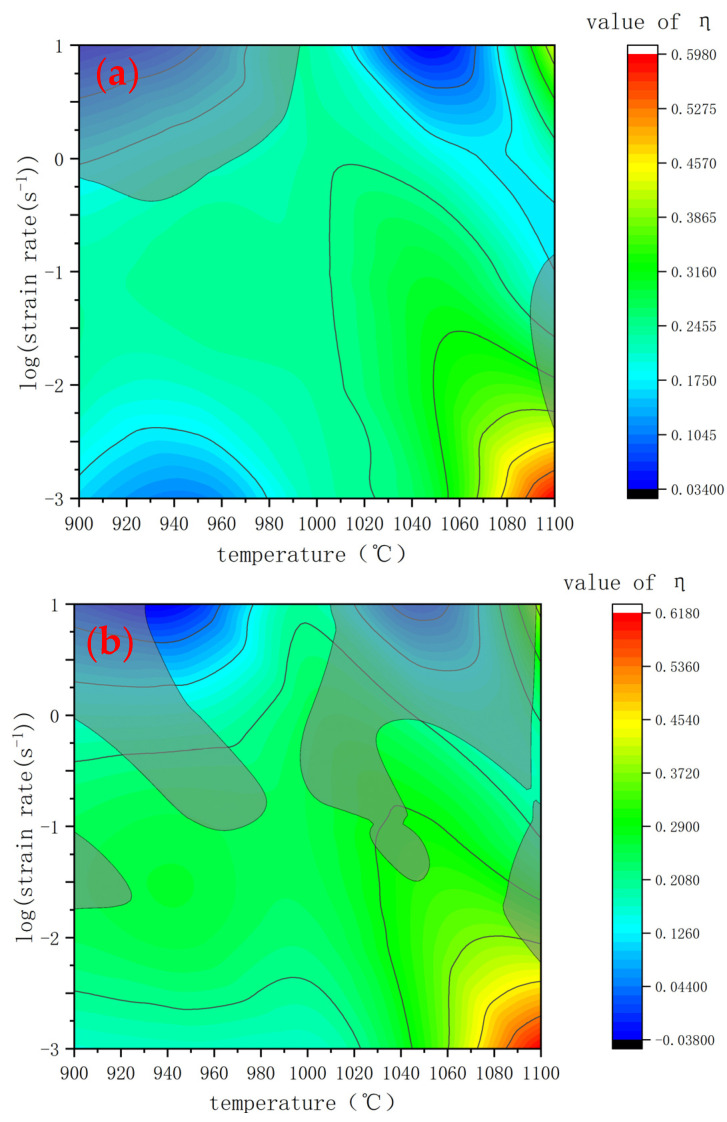

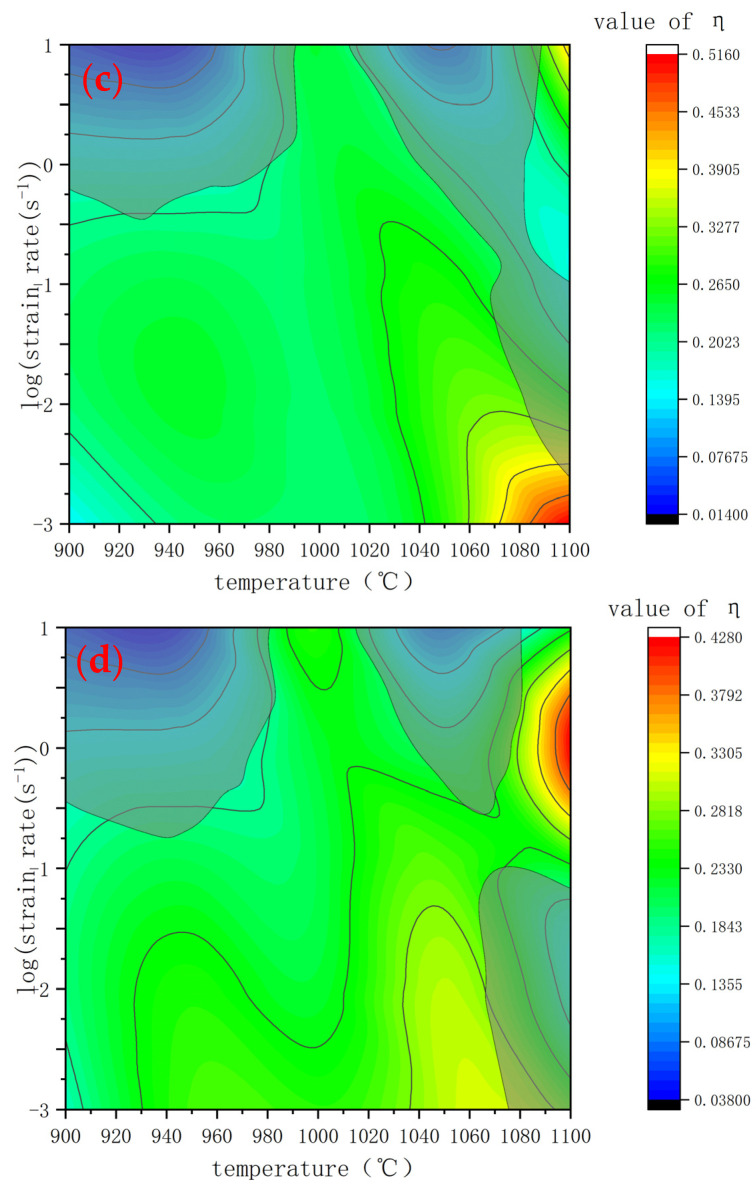
Thermal processing chart under different true strains: (**a**) 0.1, (**b**) 0.2, (**c**) 0.3, and (**d**) 0.4.

Figure 7 shows the thermal processing maps under different true strains. The shaded parts represent the instability zone, and the contour line represents the energy dissipation efficiency factor (η). Generally, when η is more than 0.3, the material should have a better performance under this thermal processing parameter. As shown in Figure 7, there presents a wide range of an instability zone in all true strain ranges (0.1~0.4). Most instability zones are located in the region with a strain rate higher than 0.1 s^−1^. When the deformation temperature is above 1000 °C and the deformation rate is 0.1–10 s^−1^, there are some defect areas, indicating that the experimental steel is sensitive to high temperatures and high strain rates. When the deformation temperature ranges from 900 °C to 950 °C and the deformation rate ranges from 0.1 to 10 s^−1^, there are also instability zones at this low deformation temperature, indicating that the deformation rate has a greater impact on the instability zone under this strain condition. Finally, by observing and analyzing Figure 7, we came to the conclusion: the processing parameters with 1050 °C and a strain rate of 0.001 s^−1^ can ensure the stability, low deformation resistance, and the highest energy dissipation efficiency. However, the strain rate in an actual production is about 1 s^−1^, considering the time benefit. Even so, according to the maps above, the temperature can be controlled at 985–1000 °C to prevent instability at this deformation rate. 

### 3.4. Microstructural Characteristics and Evolution

**Figure 8:**
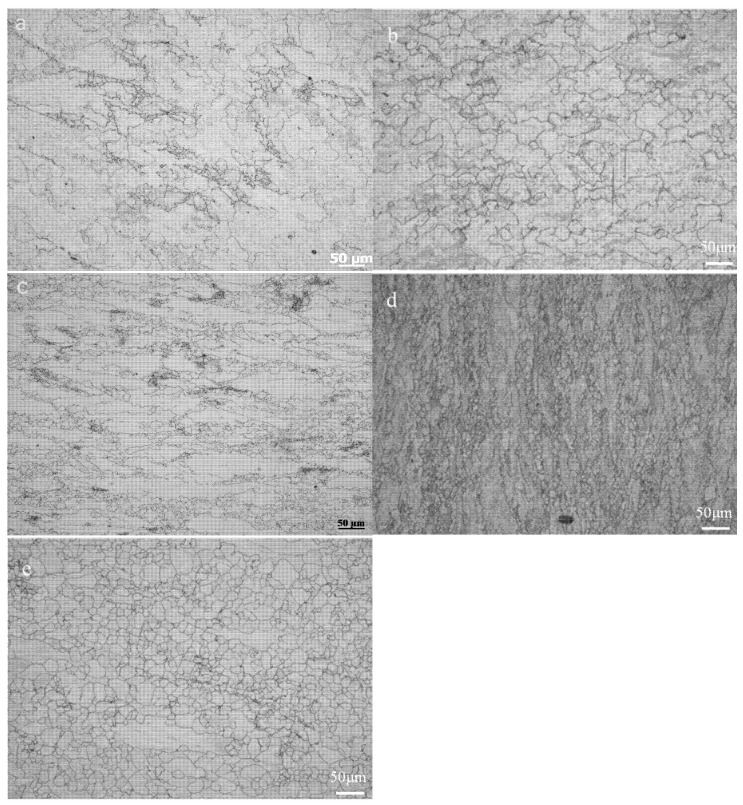
Microstructures at a deformation temperature of 1050 °C and at different deformation rates: (**a**) 0.001 s^−1^, (**b**) 0.01 s^−1^, (**c**) 0.1 s^−1^, (**d**) 1 s^−1^, and (**e**) 10 s^−1^. Average grain sizes: (**a**) 200 μm, (**b**) 110 μm, (**c**) 80 μm, (**d**) 50 μm, and (**e**) 20 μm.

Figure 8 shows optical microstructures at different deformation rates when the deformation temperature is 1050 °C. The strain rate has a significant effect on the grain size. The grain size decreases when the strain rate is from 0.001 s^−1^ to 10 s^−^^1^. As shown in Figure 8a, the grain size is coarse at the strain rate of 0.001. At this case, the deformation time is longer, and the microstructure recovery is more sufficient. Therefore, there are coarse grains in the microstructure. With the increase of the strain rate, the grain size gradually decreases (Figure 8b–e). This can be attributed to the dynamic recovery and recrystallization process during deformation, because the time to obtain the same strain decreases with the increase of the strain rate, which can greatly reduce the occurrence of dislocation recovery during the deformation. As shown in Figure 8e, the grain size is fine and equiaxed at the strain rate of 10 s^−1^. In this case, the number of recrystallization nucleation increases, and the deformation time is short. Therefore, the grains cannot grow up fully, and there is fine and equiaxed grains in the microstructure. 

It is necessary to add that, at a deformation temperature of 1050 °C and deformation rate of 0.001 s^−1^, the average particle size of the original austenite grain is about 200 μm. According to GB/T 4237-2015, there are no requirements for the grain size of the 50Cr15MoVCu steel plate. Since there are many subsequent processes, such as aging and annealing, the grain size will be greatly improved.

**Figure 9:**
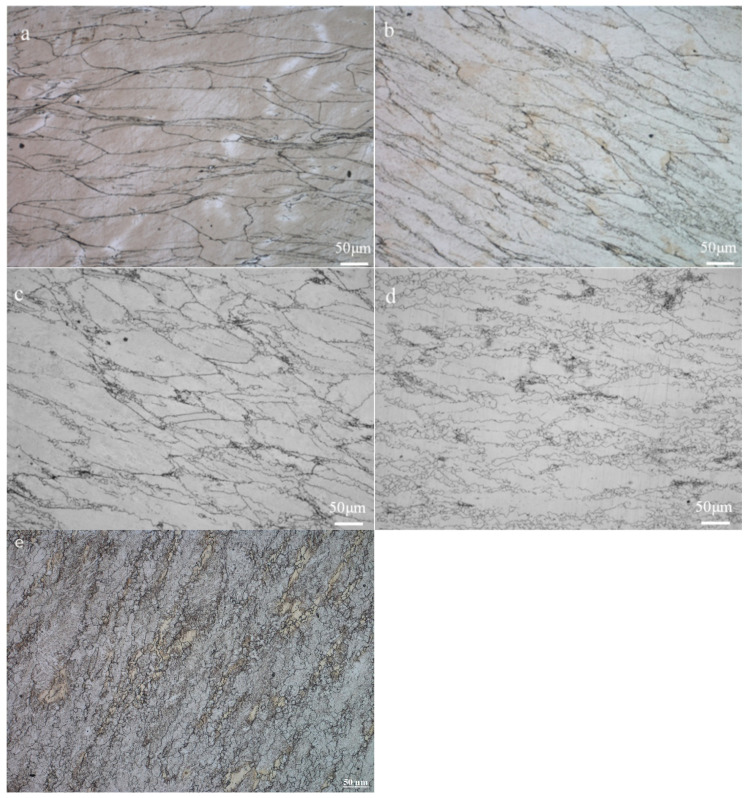
Microstructure at a deformation rate of 0.1 s^−1^ and at different deformation temperatures: (**a**) 900 °C, (**b**) 950 °C, (**c**) 1000 °C, (**d**) 1050 °C, and (**e**) 1100 °C. Average grain lengths: (**a**) 310 μm, (**b**) 230 μm, (**c**) 150 μm, (**d**) 110 μm, and (**e**) 70 μm.

Figure 9 shows optical microstructures at different deformation temperatures when the deformation rate is 0.1 s^−1^. At the deformation rate of 0.1 s^−1^ and the deformation temperature of 900 °C, the obtained microstructure is elongated, as shown in Figure 9a. The mechanism attributed to the prior austenite grain tends to elongate along the deformation direction. With the increase of the deformation temperature, the long-banded microstructure becomes fine but still distributes along the deformation direction, and a large number of recrystallization nucleation occurs (Figure 9b–e). When the deformation temperature is low, a dislocation movement is relatively difficult. According to the true stress–strain curve, there is mainly the recovery stage, and thus, work-hardening plays a leading role, and the microstructure elongates along the deformation direction. When the deformation temperature further increases and reaches 1100 °C, work-hardening and dynamic softening occur simultaneously, and the prior austenite grain tends to be equiaxed. However, work-hardening still plays a dominant role, and the microstructure is not fully equiaxed and only becomes fine.

## 4. Conclusions

1. The flow stress curves have three typical characteristics: (i) a single peak dynamic recrystallization curve, (ii) a monotone incremental work-hardening curve, and (iii) the equilibrium dynamic recovery curve.
2. The flow stress increased with the increase of the deformation rate at each deformation temperature and decreased with the increase of the deformation temperature at the same deformation rate.
3. The high temperature constitutive equation is:

(21) $$Z=\;\dot{\varepsilon }\exp\left(Q/\mathrm{RT}\right)=3{.42\;\times \;10}^{17}{\left[\sinh\left(0.0068\;\times \;\sigma \right)\right]}^{5.6807}$$

4. Processing parameters with 1050 °C and a strain rate of 0.001 s^−1^ can ensure the highest energy dissipation efficiency without an instability phenomenon.

## Figures and Tables

**Table 1:** Elemental composition of 50Cr15MoVCu.

Element	C	Si	Mn	Cr	Mo	V	Ni	Cu	P	S	Fe
**wt%**	0.46	0.48	0.51	15.02	0.66	0.12	0.12	3.52	0.007	0.0045	Bal.

## Data Availability

All data included in this study are available upon request by contact with the corresponding author (zyang@csu.edu.cn).
